# Quality of life and objective outcome assessment in women with tape division after surgery for stress urinary incontinence

**DOI:** 10.1371/journal.pone.0174628

**Published:** 2017-03-27

**Authors:** Daniela Ulrich, Vesna Bjelic-Radisic, Anna Höllein, Gerda Trutnovsky, Karl Tamussino, Thomas Aigmüller

**Affiliations:** Department of Obstetrics and Gynecology, Medical University of Graz, Graz, Austria; Massachusetts General Hospital, UNITED STATES

## Abstract

**Background:**

Midurethral tapes may cause long-term complications such as voiding dysfunction, groin pain, de novo urgency or mesh erosion, which necessitate a reoperation. There is a paucity of data regarding health related quality of life in patients undergoing tape removal. The aim of the study was to evaluate quality of life (QoL) and objective outcome after midurethral tape division or excision.

**Methods:**

All patients who underwent a midurethral tape division for voiding difficulties, pain or therapy resistant de novo overactive bladder between 1999 and 2014 were invited for follow-up. A control group with a suburethral tape without division was established in a 1:2 ratio and matched for age, tape used and year of tape insertion. Patients completed the Kings´ Health Questionnaire (KHQ), Incontinence Outcome Questionnaire, Female Sexual Function Index Questionnaire and the Patient Global Impression of Improvement score.

**Results:**

Tape division or excision was performed in 32 women. Overall, 15 (60%) of 25 women who were alive were available for clinical examination and completed the questionnaires. Tape division was performed for voiding dysfunction (n = 7), overactive bladder (n = 2), mesh extrusion (n = 3) and ongoing pain (n = 3). Median time to tape division/excision was 10 months. Three women in the tape division group had undergone reoperation for stress urinary incontinence (SUI). At a median follow-up of 11 years (IQR 9–13) subjective SUI rate was 53% (8/15 women) in the tape division group and 17% (5/30) in the control group (p = 0.016), with no significant differences in objective SUI rates between groups. With regard to quality of life, the study group had significantly worse scores in the SUI related domains role limitation, physical limitation, severity measures and social limitations (KHQ) compared to the control group.

**Conclusions:**

Women needing tape division or excision have lower SUI related QoL scores compared to controls mostly because of higher subjective SUI rates.

## Introduction

Permanent midurethral tapes inserted through the vagina have become a mainstay of the surgical treatment for stress urinary incontinence (SUI) [1,2]. The first retropubic tape was described by Ulmsten et al. in 1996 and was soon available commercially as the tension-free vaginal tape (TVT) [3]. Nine years later, a transobturator tape was introduced by de Leval in 2005 [4]. Today a number of retropubic and transobturator systems are available and the literature is considerable [5]. The reoperation rate after tape placement for urinary retention, voiding dysfunction, recurrent urinary tract infection, mesh erosion or pain has been reported to be 2.7% [6]. Complications after midurethral tapes include voiding dysfunction, groin pain, infection, necrotizing fasciitis and chronic problems like de novo urgency, and sling erosion/extrusion [7]. Voiding dysfunction is reported between 4% and 7% depending on the tape used [8]. Sling loosening can be performed within 7–10 days after surgery with low complication rates and high success rates in terms of SUI [9–12]. Spontaneous resolution may occur up to 6 weeks [13]; for persistent voiding dysfunction beyond this, a formal transvaginal sling lysis or removal is necessary due to fibroblast invasion [7]. The longer the time between the onset of problems and the intervention the higher is the rate of irreversible bladder symptoms [14].

Validated questionnaires are reliable and valid measurements of subjective outcomes. Both the International Urogynecologic Association (IUGA) and the International Continence Society (ICS) recommend including quality of life (QoL) as an outcome in clinical research [15]. There is a paucity of data regarding QoL in patients undergoing tape loosening or removal. The aim of the present study was to evaluate QoL in women undergoing tape division or removal after midurethral tape for SUI. Secondary aims were to analyze objective outcome, patient satisfaction, and sexual health.

## Methods

This is a retrospective study of women who had to undergo tape division or removal for voiding dysfunction, overactive bladder and ongoing pain between 1999 and 2014. A control group of women with a suburethral tape without revision surgery was established in a 1:2 ratio and matched for age, tape used and year when the tape was inserted. Women were identified using the hospital database. The study was approved by the local ethics committee and all participants gave written informed consent.

Preoperative clinical and demographic information were abstracted from the clinic charts. The tapes were performed as described by Ulmsten [3] and de Leval [4], with or without concomitant surgery. Procedures were performed by five attending surgeons during the study period. The retropubic tape used was the Tension-free Vaginal Tape (Gynecare, Somerville, NJ), the transobturator tape used was the TVT-O (Gynecare, Johnson & Johnson), the TVT- S tape used was the TVT Secur (Gynecare, Ethicon). For revision, the tape was either solely cut suburethrally or excised in the accessible parts under the pubic ramus ranging from 5-15mm to both sides.

To evaluate QoL, subjective or objective SUI rates and sexual health women were contacted via mail and asked to attend our clinic for a standardized urogynaecological examination and to fill out questionnaires. Minimum follow-up was 12 months since tape division.

QoL was assessed with validated questionnaires including the King’s Health Questionnaire (KHQ) and the Incontinence Outcome Questionnaire (IOQ). Sexual health was evaluated using the Female Sexual Function Index (FSFI) questionnaire. The KHQ is a validated 32-item questionnaire in women with stress urinary incontinence and assesses the impact of incontinence on QoL. Lower scores indicating better QoL [16]. The IOQ was specifically designed for outcome evaluation after midurethral sling operations and is validated for postoperative assessment of QoL after surgical treatment for SUI. Higher scores indicate worse treatment outcome [17]. The FSFI is a validated 19-item questionnaire for assessment of female sexual function. Higher scores indicate better sexual function [18]. The Patient Global Impression of Improvement questionnaire (PGI-I) is a validated tool to assess the response to an intervention and was used in its published version, i.e. assessment of lower urinary tract symptoms (LUTS) [19].

Evaluation included a comprehensive history with all succeeding operations and urogynecological examination, assessment of residual urine, urodynamics (cystometry, midurethral closure pressure), a standardized cough stress test, and cystoscopyin case of overactive bladder. Objective SUI was defined as a positive cough stress test at bladder filling of 300 ml. Subjective SUI was defined when patients responded “yes” to the question: “Does urine leak when you are physically active, exert yourself, cough, or sneeze?”. All women were asked about postoperative voiding difficulties, ongoing groin pain, and de novo or ongoing urgency symptoms.

Patients not available for physical examination at the clinic underwent a telephone interview covering overall and disease-specific history and returned the above mentioned questionnaires.

Statistical analysis was performed with SPSS. Differences between the groups were analyzed by means of the chi square test or Fisher’s exact test for categorical variables and the t-test for independent samples for numerical variables. Differences between the groups in parity were analyzed by means of the Mann-Whitney-U-test. In case of heterogeneous variances, the correction by Welch was computed. Relations between the time since surgery and revision, revision and follow-up and QoL were examined by means of the Spearman rank correlation. A partial correlation between years since revision and QoL, controlling for OAB, was carried out. A logistic regression analysis was done between subjective and objective cure as the dependent variables and the time since surgery and revision and revision and follow-up, respectively. If no parameter estimation was possible, a point-biserial correlation was carried out instead. There was no external funding for this study.

## Results

**Fig 1:**
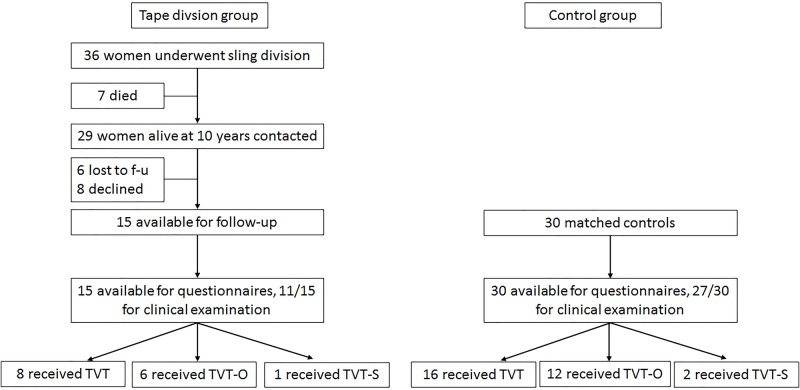
Flow chart showing study population.

Between 1999 and 2014 622 suburethral tape operations were performed at our department with 32 women needing tape division (5%). At the time of follow-up seven women had died, six were lost to follow up, eight declined participation thus leaving 15 patients for the study population (Fig 1). Eight patients had received a TVT, six a TVT-O and one patient a TVT-S; in the control group (30 women) 16 women had received a TVT, twelve a TVT-O and two a TVT-S. Baseline characteristics of the study population at the time of tape insertion are summarized in Table 1. There were no significant differences between women needing tape divisions and controls. Preoperatively, five women in the tape division group (33.0%) and five women (16.7%) in the control group reported overactive bladder (OAB) symptoms with none of them having detrusor overactivity (DO) on urodynamics.

**Table 1:** Demographic data of patients at the time of incontinence surgery.

	Tape division group (n = 15)	Control group (n = 30)	p- value
Age (years)	60.20 ± 7.51	57.43 ± 4.41	.126
BMI (kg/m^2^)	30.65 ± 6.47	29.74 ± 7.58	.702
Menopausal status			.542
Premenopausal	1 (6.7%)	3 (10.0%)	
Postmenopausal	7 (46.7%)	18 (60.0%)	
HRT	7 (46.7%)	9 (30.0%)	
Previous surgery			.838
Hysterectomy	3 (20.0%)	4 (13.3%)	
POP surgery	3 (20.0%)	7 (23.3%)	
Anti-incontinence procedures (Burch)	1 (7.0%)	1 (3.0%)	
Concomitant surgeries			0.168
Vaginal hysterectomy	2 (13.3%)	2 (6.7%)	
Vaginal hysterectomy + colporrhaphy	2 (13.3%)	4 (13.3%)	
Colporrhaphy only	2 (13.3%)	0	
Hysteroscopy	1 (7.0%)	2 (7.0%)	
Adnexectomy	1 (7.0%)	0	
POP-Q			
Ba	-1.33 ± 0.97	-1.48 ± 1.77	.823
Bp	3.50 ± 0.71	3.21 ± 3.50	.456
C	-4.50 ± 1.98	-4.69 ± 2.55	.865
Pads usage per day	2.33 ± 2.00	3.63 ± 2.10	.114
Residual urine (ml)	71.25 ±13.12 111.68	8.67 ± 18.47	.344
Detrusor contractions	0 (0%)	1 (4.2%)	1.000
MUCP (cm H_2_O)	47.00 ± 12.53	52.47 ± 30.62	.654

Data are expressed as number (%) or mean ±SD or median (range). BMI, body mass index; HRT, hormonal replacement therapy; POP-Q, pelvic organ prolapse quantification; MUCP, midurethral closure pressure.

Tape division or excision was performed for voiding dysfunction (n = 7), OAB (n = 2), mesh extrusion (n = 3) and ongoing pain (n = 3). Median time to tape division or excision was 10 months (range 1–71). In 8 cases the tape was excised, in 7 cases a simple tape division was performed. Median follow-up for the study examination was 11 (range 1–15) years for the tape division group and 11 (1–16) years for the control group since the initial operation; and 10 (1–15) years since the tape was divided or excised. Patients’ characteristics at follow-up are detailed in Table 2.

**Table 2:** Clinical data at time of follow up.

	Tape division group (n = 15)	Control group (n = 30)	p- value
Age	71 (± 10)	70 (± 5)	.655
Smoker			.348
Yes	3 (25%)	3 (11.1%)	
No	9 (75%)	24 (88.9%)	
Sexual activity			.710
Yes	11 (73.3%)	24 (80.0%)	
No	4 (26.7%)	6 (20.0%)	
UTI within last year			.287
Yes	5 (35.7%)	6 (20%)	
No	9 (64.3%)	24 (80%)	
Pad usage			.029
Yes	11 (73.3%)	11 (36.7%)	
No	4 (26.7%)	19 (63.3%)	
Hormonal status			.259
Premenopausal	1 (6.7%)	0 (0%)	
Postmenopausal without HRT	12 (80.0%)	28 (93.3%)	
Postmenopausal on HRT	2 (13.3%)	2 (6.7%)	
Reoperation			
For incontinence	3 (20.0%)	0 (0%)	.032
For gynecological reasons	1 (6.7%)	3 (10.0%)	1.000
Time since initial surgery (years)	11 (1–15)	11 (1–16)	1.000
Time between intitial surgery and division (months)	10 (1–71)	n.a.	n.a.
Time since tape division (years)	10 (1–15)	n.a.	n.a.
Subjective cure of SUI			.016
Yes	7 (46.7%)	25 (83.3%)	
No	8 (53.3%)	5 (16.7%)	
PGI-I for LUTS			.051
improved	7 (46.7%)	21 (77.8%)	
no change	4 (26.7%)	1 (3.7%)	
worsened	4 (26.7%)	5 (18.5%)	

Data are expressed as number (%), mean ±SD or median (range). HRT, hormone replacement therapy; UTI, urinary tract infection; SUI, stress urinary incontinence; PGI-I, Patient Global Impression of Improvement questionnaire; LUTS, lower urinary tract symptoms.

QoL and sexual health results are shown in Tables 3–5. With regard to QoL, women in the tape division group showed significantly worse scores in most SUI related domains like role limitation, physical limitation, severity measures and social limitations compared to the control group (Table 3). No significant differences were seen for non SUI related domains like personal relationship, sleep, emotions, recurrent UTI and painful or overactive bladder (Table 3). The extended score of the IOQ questionnaire revealed significantly worse results in the tape division group but the only significant subdomain was hospital readmission (Table 4). Sexual function as determined with the FSFI showed a significantly better total score in the control group. While the subdomains lubrication, orgasm and pain were significantly better in the control group there were no differences for desire, arousal or satisfaction (Table 5).

**Table 3:** Results of the Kings Health questionnaire at study visit.

	Tape division group (n = 15)	Control group (n = 30)	p- value
General health perception	48.08 ± 18.99	35.83 ± 20.43	.073
LUTS impact	51.28 ± 39.94	32.22 ± 32.14	.105
Role limitation	55.95 ± 34.96	25.29 ± 30.74	.005
Physical limitations	69.44 ± 31.65	21.11 ± 22.71	<.001
Social limitations	41.27 ± 36.57	9.07 ± 17.17	.006
Personal relationship	44.44 ± 50.92	15.74 ± 32.58	.204
Emotions	33.33 ± 33.95	14.56 ± 23.96	.089
Sleep/energy	34.72 ± 26.07	20.69 ± 27.69	.142
Severity measures	70.26 ± 30.63	44.11 ± 30.09	.013
Overactive bladder	52.88 ± 36.77	37.68 ± 26.75	.162
Leakage during activity	61.54 ± 41.60	55.56 ± 41.62	.696
Enuresis	75.00 ± 41.83	60.00 ± 54.77	.618
Leakage during intercourse	100.00 ± 0.00	75.00 ± 35.36	.180
Recurrent LUT-infection	60.00 ± 41.83	56.25 ± 41.73	.878
Painful bladder	62.50 ± 47.87	57.14 ± 34.50	.833
Voiding difficulties	41.67 ± 49.16	35.00 ± 47.43	.792

Data are expressed as mean ± standard deviation except for the p-values. (Lower scores indicate better quality-of-life.)

**Table 4:** Results of incontinence outcome Questionnaire at study visit.

	Tape division group (n = 15)	Control group (n = 30)	p- value
Extended score (QOL, satisfaction)	42.22 ± 14.39	28.73 ± 14.85	.013
Pain	14.55 ± 22.07	11.33 ± 21.45	.676
Urinary infection	38.46 ± 50.64	37.93 ± 49.38	.975
Other infection	30.77 ± 48.04	34.48 ± 48.37	.819
Hospital readmission	61.54 ± 50.64	6.67 ± 25.37	.002
Symptoms preoperative	70.83 ± 25.75	71.67 ± 30.61	.934
Overactive bladder preoperative	70.00 ± 48.30	82.14 ± 39.00	.433

Data are expressed as mean ± standard deviation except for the p-values. QoL, quality of life. (Higher scores indicate worse treatment outcome).

**Table 5:** Results of the FSFI-Questionnaire at study visit.

	Tape division group (n = 8)	Control group (n = 24)	p- value
Desire	1.65 ± 0.70	2.14 ± 0.97	.202
Arousal	0.73 ± 1.33	2.01 ± 1.92	.079
Lubrication	0.53 ± 1.38	2.19 ± 2.42	.023
Orgasm	0.00 ± 0.00	1.81 ± 2.13	.017
Satisfaction	2.40 ± .	4.00 ± 1.69	.385
Pain	0.00 ± 0.00	2.08 ± 2.66	.038
Full scale	3.00 ± 3.11	11.87 ± 10.94	.001

Data are expressed as mean ± standard deviation except for the p-values. (Higher scores indicate better sexual function.)

**Fig 2:**
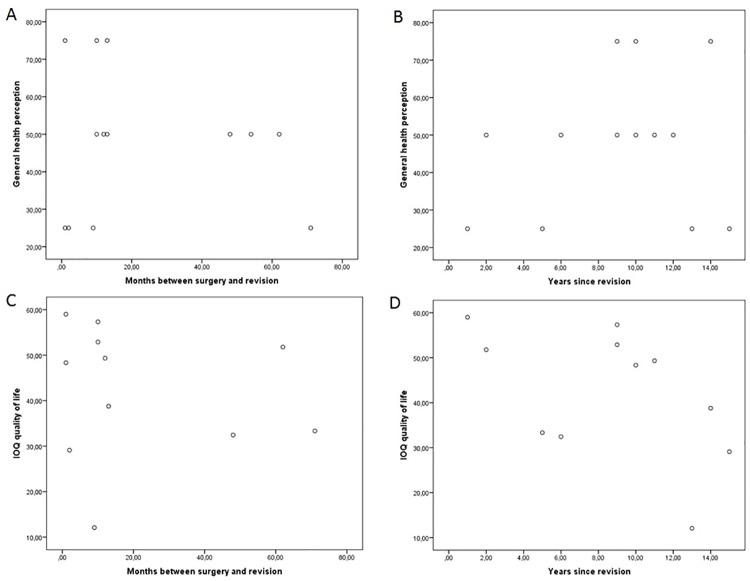
Scatter plot—Correlations between quality of life and time intervals. A: General health perception (Kings Health questionnaire) versus time interval between initial surgery and revision. B: General health perception (King Health questionnaire) versus time since revision surgery and follow-up. C: Extended QoL (Incontinence Outcome Questionnaire) versus time interval between initial surgery and revision. D: Extended QoL (Incontinence Outcome Questionnaire) versus time interval between initial surgery and revision.

At time of follow-up there was no significant correlation between general health perception (KHQ) and time interval between initial surgery and revision or time since revision surgery and follow-up (r = 0.05, p = 0.881; r = -0.15, 0.668) (Fig 2A + 2B). Neither was there any significant correlation between the IOQ extended QoL score and interval between initial surgery and revision (r = 0.11, p = 0.729), (Fig 2C). There was a trend between the IOQ extended QoL score and time since revision surgery and follow-up (r = -0.56, p = 0.073), (Fig 2D). However, after adjusting for OAB this trend could not be seen any more (r = -0.24, p = 498). After adjusting for age, no significant correlations were observed with years since revision or time between initial surgery and revision either (r = 0.50, p 0.141).

At time of follow-up, subjective SUI rate was 53% (8/15 women) in the tape division group and 17% (5/30) in the control group (p = 0.016). Objective SUI rate was 33% (4/12 women) in the tape division group and 11% (3/27) in the control group (p = 0.172) (Table 6). According to the PGI-I 53.3% (8/15) reported no improvement or no change in regard to their LUTS in the tape division group and 22% (6/27) in the control group (p = 0.051). Three women in the tape division group had undergone reoperation for incontinence (TVT, TVT-O, and Bulkamid^®^ at 1 months, 2 and 7 years after the division, respectively), with the woman with TVT and Bulkamid having subjective but not objective SUI and one women with the TVT-O having no subjective or objective SUI. No significant correlations were seen between subjective SUI rates and both time between initial surgery and revision or time between revision and follow up. For objective SUI a significant correlation was seen with time between initial operation and revision (r = 0.59, p = 0.044), but not with time between revision and follow up. Out of the 4 women with a short interval between operation and revision 3 (75%) had the tape excised for pain and no further incontinence surgery.

**Table 6:** Urogynecological assessment at follow-up (n = 38).

	Tape division group (n = 11)	Control group (n = 27)	p- value
Objective SUI (Stress test)			.172
Yes	4 (36.3%)	3 (11.1%)	
No	7 (63.7%)	24 (88.9%)	
Erosion			.341
Yes	1 (9.1%)	0 (0%)	
No	10(90.9%)	27 (100%)	
Residual urine (ml)	41 ± 91	18 ± 24	.390
MUCP (cm H2O)	31 ± 16	48 ± 34	.199
POP-Q			
Ba	-2.7 ± 0.7	-1.4 ± 1.1	.002
Bp	-2.5 ± 0.9	-2.4 ± 0.8	.620
C	-5.8 ± 1.5	-3.9 ± 3.8	.137

Data are expressed as absolute number (%) or mean ±SD. MUCP, midurethral closure pressure; POP-Q, pelvic organ prolapse quantification.

In the two cases with tape division for OAB, OAB symptoms did not resolve but there was also no recurrent SUI. In the three cases with persistent tape-related pain, the tape was excised up to 15mm to both sides with subsequent pain resolution. One of these patients had preoperative OAB that resolved postoperatively; recurrent SUI was present in one patient.

At follow-up 53.3% (8/15) reported symptoms of OAB in the tape division group with only two having detrusor overactivity (DO) on urodynamics; 40.0% (12/30) reported OAB in the control group without a significant difference. The OAB rate preoperatively was 33.3% (5/15) in the tape division group and 16.7% (5/30) in the control group. At time of follow up 40.0% (4/10) had de novo OAB and one woman had symptom resolution in the tape division group. In the control group 32.0% (8/25) had de novo OAB and one woman had symptom resolution.

## Discussion

In our series, patients undergoing tape division for voiding dysfunction, ongoing pain or OAB had worse outcome in regard to overall QoL compared to women without revision surgery. These differences were predominantly seen in SUI related domains due to higher subjective and objective SUI rates.

At a median follow-up of 11 years (IQR 9–13) women undergoing tape division had 53% subjective and 33% objective SUI rates. This was significantly lower than in the control group without tape division or excision (17% subjective SUI and 11% objective SUI). 53% of patients felt unchanged or worsened according to the PGII with respect to their LUTS which was also worse compared to the control group (22%). Results for both subjective and objective SUI rates in the control group are comparable to previously published long-term follow up [20,21].

Few groups have looked at outcomes in patients with tape division. Leng et al described a strong association between persistent bladder symptoms and greater delay to urethrolysis in women with ongoing bladder outlet obstruction after midurethral tape[14]. George et al examined differences in recurrence rates after urethrolysis/sling revision between normal- and overweight women [22]. In both groups the recurrence rate of SUI was around 50% which is similar to our results.

Subjective outcome reporting tools are necessary to evaluate women’s perception of QoL and sexual health. Available studies focus on reasons for tape associated risks and reoperations in the primary setting [6,7] but not on the actual subjective and objective clinical situation.

In our study, several domains in regard to QoL were significantly worse in the tape division group compared to the control group. In line with higher subjective and objective SUI rates women with tape division had worse scores for most SUI related domains including impairment of LUTS in their daily lives. In contrast, domains like recurrent UTIs or painful or overactive bladder symptoms were similar between the two groups. This may indicate that the revision surgery had relieved the initial problems causing the reoperation. Worse scores were also seen between women with and without tape division/excision in regard to their overall sexual health. However, important domains for sexual satisfaction like desire, arousal or satisfaction were similar between the two groups suggesting that tape division is an option for women with tape related issues.

Interestingly, only 20% (3/15) underwent reoperation for SUI despite the modest satisfaction. This may indicate that, women with postoperative complications are doubtful about undergoing another repeat procedure, or that treating physicians are hesitant to recommend it.

The time between tape insertion and tape excision or division was quite long compared to other reports. No differences were seen in the subjective cure rates dependent on the time of tape division/excision. There was a correlation between objective SUI and time between initial surgery and revision; however they were mostly cases who had the tape excised for pain without any further incontinence surgery. In this study various reasons for tape division/ excision were included. Ongoing pain or OAB were treated conservatively for quite a long time before a repeat surgery was indicated.

There is a relatively large discrepancy between subjective and objective SUI rates possibly related to the high OAB rate in this group or to the strict definition of subjective SUI.

We saw no erosions in the women who attended the clinic, which is less than previously reported in other long-term-follow-up studies [20]. However, one intra-urethral and two vaginal erosions had already been cured in the course of the tape excision at 2, 9 and 12 months. Furthermore, 8 women had their tape partially removed and the remaining study group is too small to draw any conclusions in this regard.

The patients in our study had a 47% OAB rate at a median follow up of 11 years, which is in line with our long-term follow-up after a single TVT-O [20]. Some of these women might have developed de novo OAB due to age. Another explanation could be that women with problems were more likely to attend the study visit or a clinical overestimation by the patients themselves since only 2 showed DO on urodynamics.

The strength of our study is the presentation of subjective and objective outcomes including assessment of QoL, clinical examination and evaluation of sexual health. Limitations of the study are the absence of preoperative QoL data from our patients and the small sample size. The relatively high lost to follow-up rate may be explained by the long study period; the high number of deceased patients is likely due to the advanced age at initial surgery. A further weakness of the study is the high range in the follow-up interval. However, there were no differences in several QoL parameters dependent on the timeframe since revision surgery.

## Conclusions

In conclusion, women after tape division/ excision have lower QoL scores compared to controls mostly because of higher subjective SUI rates.
